# Genetic diversity of *Staphylococcus aureus* strains from a teaching hospital in Isfahan, Iran: The emergence of MRSA ST639- SCC*mec* III and ST343- SCC*mec* III

**Published:** 2018-04

**Authors:** Hossein Sedaghat, Bahram Nasr Esfahani, Mehrdad Halaji, Azhar Sallari Jazi, Sina Mobasherizadeh, Seyed Rohollah Havaei, Mohammad Emaneini, Seyed Asghar Havaei

**Affiliations:** 1 Department of Microbiology, School of Medicine, Isfahan University of Medical Sciences, Isfahan, Iran; 2 Nosocomial Infection Research Center, Isfahan University of Medical Sciences, Isfahan, Iran; 3 Department of Endodontics, Dental School, Khorasgan University, Isfahan, Iran; 4 Department of Microbiology, School of Medicine, Tehran University of Medical Sciences, Tehran, Iran

**Keywords:** MRSA, MLST, Nasal carrier, SCC*mec* typing

## Abstract

**Background and Objectives:**

*Staphylococcus aureus* is an important pathogen that can be colonized in the nose and increase the risk of spreading infections in hospitals. The present study aimed at determining the phenotypic and genotypic characterization of *S. aureus* strains isolated from patients and healthcare workers (HCWs) from a teaching hospital in Isfahan, Iran.

**Materials and Methods:**

This cross-sectional study was performed on 262 nasal swabs and 23 clinical isolates that were collected from a teaching hospital during February and April 2016. Staphylococcal cassette chromosome *mec* (SCC*mec*) and multilocus sequence typing (MLST) were performed for selected isolates.

**Results:**

Overall, 23% and 18% of healthcare workers and patients were carriers, respectively. Methicillin-resistant *S. aureus* (MRSA) rate was 13%, 33% and 52% in nasal HCWs, nasal patients, and clinical samples, respectively. The molecular typing of MRSA isolates revealed that the most common SCC*mec* type is SCC*mec* type III (88%). The highest rate of resistance was observed against tetracycline and erythromycin, with 48.7%. The most frequently detected toxin genes among *S. aureus* isolates were *hla* (99%) and *sea* (44%), moreover, *pvl* genes were detected in (40%) of MRSA isolates. The results of MLST showed 7 different sequence types (STs): ST859 (2/9), ST6 (2/9), ST639 (1/9), ST343 (1/9), ST239 (1/9), ST291 (1/9) and ST25 (1/9).

**Conclusion:**

Our results revealed that ST clones associated with healthcare-associated MRSA (HA-MRSA) are actively circulating among nasal carriage in our healthcare setting, and thus, effective infection control policies are needed to reduce nasal carriage in healthcare settings.

## INTRODUCTION

*Staphylococcus aureus* is a significant pathogen causing different infectious diseases that can become a life-threatening pathogen. The nose is the most common carriage site for *S. aureus* and nasal carriers are at risk of developing *S. aureus* infections. In recent years, the increasing number of infections caused by methicillin-resistant *S. aureus* (MRSA) and resistance to various antibiotics have been a great challenge for clinicians (1, 2). The acquisition of *mec*A is responsible for changing methicillin-susceptible *S. aureus* (MSSA) to methicillin-resistant *S. aureus*. Staphylococcal cassette chromosome *mec* (SCC*mec*) is a unique mobile genetic element that carries the *mec*A gene. So far, 11 main types of SCC-*mec* have been identified, which vary in size and genetic content, moreover, these elements contain multiple resistance genes. Most of healthcare-associated MRSA (HA -MRSA) strains carry SCC*mec* types, I, II, and III, whereas SCC*mec* types IV and V have been usually associated with community-associated MRSA (CA-MRSA) (3).

It is well documented that pathogenesis of *S. aureus* relates to various virulence factors. One of the mechanisms of pathogenesis is adherence to components of the extracellular matrix of the host. Virulence factors involved in this process are microbial surface component recognizing adhesive matrix molecules (MSCRAMMs), such as fibronectin binding proteins (FnbA and FnbB), collagen binding protein (Cna), elastin binding protein (EbpS), laminin binding protein (Eno), and fibrinogen binding protein (Fib). In *Staphylococcus* spp, intracellular adhesion (ica) locus, composing of the genes *ica*ADBC, is responsible for cell aggregation and biofilm accumulation. The *ica* locus encodes crucial proteins to produce polysaccharide intercellular adhesion (PIA) and capsular polysaccharide/adhesion (PS/A). Other virulence factors in *S. aureus* are superantigens including staphylococcal enterotoxin (SE) groups (SEA, SEB, SEC, SED and SEE) and toxic shock syndrome Toxin 1 (TSST-1), haemolysins (alpha, beta, gamma and delta), and leukocidin family, such as Panton-Valentine leukocidin (PVL) (4–7). The prevention and control strategies of *S. aureus* infections depend on analysis of the isolates by various molecular typing techniques (8). Pulsed-field gel electrophoresis (PFGE), multilocus sequence typing (MLST), and SCC*mec* typing are the most reliable typing methods. Among them, PFGE is considered the gold standard for molecular typing of MRSA and investigation of nosocomial outbreaks (9, 10). In addition, MLST typing is based on the determination of allelic profile or sequence type (ST) of internal fragments of 7 housekeeping genes and can be used to determine evolutionary and population biology of bacterial species (9). Therefore, the present study aimed at determining antimicrobial resistance profile, the prevalence of genes encoding virulence factor, MSCRAMM, and biofilm and molecular characteristic of *S. aureus* strains isolated from patients and healthcare workers from a teaching hospital in Isfahan, Iran.

## MATERIALS AND METHODS

### Study design and setting.

This cross-sectional study was conducted at a major teaching hospital (Alzahra) in Isfahan February and April 2016. This survey was in accordance with the declaration of Helsinki, and written informed consent was obtained from hospitalized patients and healthcare workers (HCWs).

A total of 262 non-duplicated nasal swabs were collected from 99 healthcare workers and 163 patients in Alzahra hospital. Among the nasal samples, 55 *S. aureus* isolates were collected and enrolled in the study. Furthermore, 23 non-duplicated *S. aureus* isolates were taken from clinical samples. A total of 78 *S. aureus* isolates were collected from clinical samples and nasal swabs of hospitalized patients and healthcare workers. Samples were collected from different wards including intensive care units (ICUs), internal medicine, infectious, emergency, surgery, and operating room. In addition, clinical isolates were obtained from the blood, wound, urine culture, sputum, peritoneum and synovial samples.

### Bacterial isolation and identification.

Nasal sampling was performed by rotating a sterile swab soaked with saline in the vestibule of both the personnel and patient’s nares and inoculated into mannitol salt agar medium. The nasal and clinical samples were cultured on blood agar (Merck, Germany), and plates were incubated at 37° C for 24 hours after which the colonies were recognized as *S. aureus* using such standard microbiology methods as colony morphology, Gram staining, catalase test, coagulase test, mannitol fermentation, and DNase test (11).

### Antimicrobial susceptibility testing.

The susceptibility patterns of MSSA and MRSA strains were characterized by Kirby-Bauer disc diffusion test according to the CLSI guideline (12). The panel of antibiotics (MAST, United Kingdom) used in susceptibility testing included ciprofloxacin, linezolid, chloramphenicol, trimethoprim-sulfamethoxazole, quinupristin-dalfopristin, mupirocin, teicoplanin, tetracycline, rifampin, gentamicin, erythromycin, and clindamycin. MICs for vancomycin were determined by agar dilution method according to the CLSI guideline. ATCC25923 was used as the quality control strain. All the MRSA isolates were identified by amplification of *mec*A gene by PCR method as described previously (3).

### DNA extraction and detection of *fem A*, MSCRAMMs, and *ica* genes.

DNA was extracted from the staphylococcal isolates using the method described by Ito et al. (3); *fem A* was detected by the method as described by Mehrotra et al. to confirm the presence of *S. aureus* (4). PCR amplification was performed by polymerase chain reaction (PCR) to detect adhesive surface and *ica* genes (*fnbA, fnbB, fib, eno, cna, ebps, icaA* and *icaD*) using the primers and conditions described by Vancraeynest et al. (13).

### Identification of toxin genes.

PCR amplification was performed to detect toxic shock syndrome toxin and enterotoxins (*tst, sea, seb, sec, sed)*, alpha haemolysin (*hla*), and Panton-Valentine leukocidin toxin (*pvl*) genes using the primers and conditions described by Johnson et al., Jarraud et al., and sauer et al., respectively (14–16).

### SCC*mec* typing.

Multiplex-PCR (MPCR) was performed to identify SCC*mec* types as described previously (3). MPCR-1 was done to detect *mec*A and *ccr* genes, whereas M-PCR-2 was performed to determine *mec* gene complex classes A, B and C2.

### Multilocus sequence typing.

A total of 9 isolates, 7 MRSA isolates and 2 MSSA isolates, were subjected to MLST (http://sepidermidis.mlst.net/). STs of isolates were detected using the *S. aureus* MLST database, and their CCs were determined using the eBURST algorithm.

Selection of MRSA isolates for MLST was performed based on SCC*mec* type and antibiotic resistance patterns, whereas MSSA was selected randomly. The housekeeping genes of each MRSA isolate were amplified and sequenced as described previously by Enright et al. (9).

### Statistical analysis.

Data were analyzed using SPSS^TM^ software Version 21.0 (IBM Corp., USA). The results are presented as descriptive statistics in relative frequency. Chi-square or Fisher’s exact tests were used to determine the significance of the differences. A difference was considered statistically significant if the *p*-value was less than 0.05.

## RESULTS

Of the 78 confirmed *S. aureus* strains, 23 isolates which 32% (25/78) were MRSA. *S. aureus* carriage rate was 23% (23/99) and 18% (30/163) among HCWs and patients, respectively. The prevalence rate of MRSA nasal carriages among patients and HCWs was 33% (10/30) and 13% (3/23), respectively. Meanwhile of the 23 clinical *S. aureus* isolates, 52% (12/23) were identified as MRSA.

Antibiotic susceptibility pattern showed that all *S. aureus* isolates were susceptible to linezolid, quinupristin-dalfopristin, and mupirocin, while the highest rate of resistance was observed against tetracycline and erythromycin, with 48.7%. Table 1 demonstrates the complete results of antibiotic susceptibility patterns. The results of the agar dilution method demonstrated that all isolates were susceptible to vancomycin, and 5 isolates showed the lowest susceptibility to vancomycin (MIC = 2 μg/mL).

**Table 1:** Antibiotic resistance prevalence MRSA and MSSA isolates among nasal carriage and clinical samples.

**Antibiotic**	**N (%) of isolates**		**Total** **(N= 78)**

	**MSSA** **N=53**	**MRSA** **N=25**	
Chloramphenicol	3 (6)	0	3 (4)
Ciprofloxacin	6 (11)	21 (84)	27 (35)
Clindamycin	10 (19)	20 (80)	30 (38)
Erythromycin	17 (32)	21 (84)	38 (49)
Gentamycin	3 (6)	18 (72)	21 (27)
Linezolid	0	0	0
Mupirocin	0	0	0
Quinupristin-dalfopristin	0	0	0
Rifampin	3 (6)	16 (64)	19 (24)
Teicoplanin	0	1 (4)	1 (1)
Tetracycline	16 (30)	21 (84)	37 (47)
Trimethoprim-sulfamethoxazole	1 (2)	4 (16)	5 (6)

### Results of molecular typing of isolates.

The results of PCR assay revealed that the most frequent MSCRAMMs, *ica* and toxin genes in *S. aureus* isolates, with 95%, 76% and 44%, respectively. Moreover, none of the isolates contained *fnbA* genes. Our results also revealed a significant association in the prevalence rate of *pvl* gene in MRSA isolates than MSSA isolates (p = 0.0164). The prevalence rate of MSCRAMMs, *ica* and toxin genes are summarized in Table 2. The results of statistical analysis showed that the rate of *icaD* (p = 0.0009), *icaA* (p = 0.0001), *cna* (p = 0.0021), *fib* (p = 0.0089), and *sec* (p = 0.0001) was significantly higher in clinical samples than nasal samples.

**Table 2:** Prevalence of genes encoding virulence determinants of MRSA and MSSA isolates among nasal carriage and clinical samples.

**Virulence genes**	**N (%) of isolates**		**Total** **(N= 78)**

	**MSSA** **N=53**	**MRSA** **N=25**	
*hla*	52 (98)	25 (100)	77 (99)
*pvl*	7 (13)	10 (40)	17 (22)
*tst*	9 (17)	4 (16)	13 (17)
*sea*	20 (38)	14 (56)	34 (44)
*seb*	6 (11)	7 (28)	13 (17)
*sec*	11 (21)	5 (20)	16 (21)
*sed*	11 (21)	9 (36)	30 (38)
*ica*A	39 (74)	18 (72)	57 (73)
*ica*D	43 (81)	16 (64)	59 (76)
*cna*	28 (53)	19 (76)	47 (60)
*ebpS*	28 (53)	11 (44)	39 (50)
*eno*	49 (92)	25 (100)	74 (95)
*fib*	30 (57)	20 (80)	50 (64)
*fnb*A	0	0	0
*fnb*B	11 (21)	4 (16)	15 (19)

The molecular characteristics of the MRSA isolates are presented in Table 3. Based on the Multiplex-PCR analysis, most of the MRSA isolates (88%) harbored SCC*mec* type III, meanwhile 12% (3/25) of the isolates carried type I, II, and IV, each with 1 isolate. According to the MLST method, 7 different sequence types (STs) were found among 9 selected isolates. As demonstrated in Table 3, ST859 (2/9) and ST6 (2/9) were the most frequent ST types among MRSA isolates, whereas ST639, ST343, ST239, ST291, and ST25 were detected only in 1 isolate. Among them, ST 25 and ST291 belonged to MSSA clones and other STs to MRSA clones.

**Table 3:** The phenotypic and genotypic characteristics of the 25 methicillin-resistant *S. aureus* isolates from nares and clinical samples

**Isolate number**	**Ward**	**Source**	**Antibiotic resistance pattern**	**MSCRAMMs and biofilm genes**	**Toxin genes**	**SCC*mec* types**	**ST**
1	Urgency	blood	FOX, CIP, E, CD, T, RP, GM	*icaD, icaA, ebpS, eno, fib, cna*	*hla, sed*	III	639
2	ICU	trachea	FOX, CIP, E, CD, T, RP, GM	*ica D, fib, ebpS, icaA, cna, eno*	*sea, seb, sed, hla, pvl*	III	343
3	Surgery Room	wound	FOX, T	*ica D, fib, cna, eno*	*hla, sea*	III	859
4	Urgency	urgency	FOX, E	*ica D, fib, ebpS, icaA, cna, eno*	*sea, hla, pvl*	IV	6
5	Surgery	Nasal health care worker	FOX, T	*fib, fnbB, icaA, eno*	*sec, hla*	II	859
6	Surgery	Nasal Patient	FOX, CIP, T, RP, GM	*ica D, fib, cna,eno*	*hla, pvl*	III	239
7	ICU	Nasal Patient	FOX, CIP, E, CD	*ica D, fib, ebpS, cna, eno*	*sea, hla, pvl*	I	6
8	Urgency	blood	FOX, CIP, E, CD, T, RP, GM	*ica D, fib, ebpS, icaA, cna, eno*	*sea, sed, hla, pvl*	III	
9	Internal Medicine	trachea	FOX, CIP, E, CD, T, RP, GM	*ica D, fib, fnbB, ebpS, icaA, cna, eno*	*sea, sed, hla, pvl*	III	
10	Urgency	wound	FOX,CIP, E, CD, TS, T, RP, GM	*ica D, fib, ebpS, icaA, cna, eno*	*sea, sed, hla, pvl*	III	
11	Internal Medicine	wound	FOX,CIP, E, CD, T, RP, GM	*ica D, fib, fnbB, ebpS, icaA, cna, eno*	*sea, hla, pvl*	III	
12	ICU	trachea	FOX,CIP, E, CD, T, RP, GM	*ica D, fib, icaA, cna,eno*	*sea, sec, sed, hla*	III	
13	Urgency	wound	FOX,CIP, E, CD, TS, T, GM	*ica D, fib, icaA, cna,eno*	*sea, sec, sed, hla, pvl*	III	
14	Internal Medicine	trachea	FOX, CIP, E, CD, T, RP, GM	*icaD, fib, cna, eno*	*sed, hla*	III	
15	Surgery	wound	FOX, CIP, E, CD, TS, T, RP, GM	*ica D, fib, ebpS, icaA, cna, eno*	*sea, sec, sed, hla*	III	
16	ICU	Nasal health care worker	FOX	*icaA, cna, eno*	*sea, hla*	III	
17	ICU	Nasal Patient	FOX,CIP, E, CD, T, RP, GM	*fib, eno*	*hla, tst*	III	
18	ICU	Nasal Patient	FOX, CIP, E, CD, T, RP, GM	*ica D, fib, fnbB, icaA, cna, eno*	*hla, tst*	III	
19	ICU	Nasal Personnel	FOX, CIP, E, CD, T, RP,	*fib, icaA, eno*	*hla, pvl, tst*	III	
20	Surgery	Nasal Patient	FOX, CIP, E, CD, T, RP, GM	*ica D, cna, eno*	*hla*	III	
21	Surgery	Nasal Patient	FOX,CIP, E, CD,TS, GM	*cna, eno*	*sea, sec, hla*	III	
22	Surgery	Nasal Patient	FOX, CIP, E, CD, TS, T, GM	*fib, ebpS, icaA, eno*	*see, hla, tst*	III	
23	Surgery	Nasal Patient	FOX,CIP, E, CD, T	*fib, ebpS, icaA, eno, icaD,*	*hla*	III	
24	Surgery	Nasal Patient	FOX,CIP, E, CD, T, RP, GM	*icaD, icaA, cna, eno*	*sea, hla*	III	
25	Surgery	Nasal Patient	FOX,CIP, E, CD, T, RP, GM	*icaA, eno*	*seb, hla*	III	

## DISCUSSION

The prevalence of MRSA is shown to be high in the healthcare setting in Iran. Based on a recent review and meta-analyses study, the rate of MRSA was found to be 32.8% (17). In addition, the widespread emergence of MRSA nasal carriage among HCWs and patients with high resistance to different classes of antibiotics has been known as a risk factor for the development of infections (18, 19). The study of 99 HCWs from the largest teaching hospital in our region showed that the nasal carriage of *S. aureus* in this population was 23% these findings are partially consistent with those of previous Iranian studies, which ranged from 10% to 31% (20–22). The prevalence rate of MRSA colonization in nasal patients was considerably higher than nasal HCWs with 33%, which was similar to previous studies that reported higher colonization of MRSA among patients compared to HCWs (23, 24). Indeed, various factors can be attributed to this higher MRSA colonization including hospitalization in a long-term care facility, the characteristics of the population studied, and local infection control policies (25). Similar to the findings in the previous study, the SCC*mec* analysis of MRSA isolates indicated that the SCC*mec* types III were the most common SCC*mec* type, which is considerably associated with HA-MRSA. These results support that the HA-MRSA is perhaps more present than CA-MRSA in hospital environments, which is attributable to the spread of a limited number of HA-MRSA genotypes in the population studied in the healthcare setting. In the present study, antibiotic susceptibility pattern showed that the maximum resistance of MRSA isolates was against such antibiotics as ciprofloxacin (84%) and tetracycline (84%), followed by erythromycin (84%) and clindamycin (80%). As expected, the results may be due to extensive use of these antibiotics at hospitals for the treatment of different infections due to MRSA isolates. In the current study, MLST method showed 7 sequence types including ST239, ST859, ST6, ST343, ST639, ST29, and ST25, which were detected among MRSA and MSSA isolates. ST859 and ST6 were the predominant types among our isolates, with 44% frequencies. Three strains including ST859- SCC*mec* II, ST6- SCC*mec*I, and ST239- SCC*mec*III clones belonged to the nasal carrier and ST859- SCC*mec* III, ST6- SCC*mec* IV, ST639- SCC*mec* III, ST343-SCC*mec* III, ST25, and ST291 were present among different clinical isolates. In a previous study conducted by our group, it was found that isolates belonged to ST859, ST239, ST291, ST6, and ST25 had been associated with HA-MRSA isolates, which is in agreement with our finding and that of other studies in Iran (26–29). The presence of these particular ST clones in both of these studies that were performed in the teaching hospitals suggests that these ST clones are actively circulating in the healthcare setting in center of Iran (26). A notable finding of the current study was the emergence of ST343- SCC*mec*III and ST639- SCC*mec*III in Alzahra hospital. ST343- SCC*mec*III clone was isolated from trachea sample and belonged to a patient admitted to the ICU with a respiratory infection; moreover, it harbors a different virulence gene, including *sea, seb, sed, hla* and *pvl*, which was evaluated as potential virulent isolate than other isolates. Based on our results, although the 2 isolates belonging to ST859- SCC*mec*II and ST859- SCC*mec*III were isolated from nasal and wound sample, respectively, these isolates showed an exact antimicrobial susceptibility pattern and toxin profile. In this survey, the most prevalent toxin gene was *hla* (99%), which is in agreement with reports from other countries, such as the United States and Uganda (30–32). High levels of alpha-hemolysin can be associated with more severe cutaneous lesions and pneumonia (33–35). Our study indicated that 40% of MRSA isolates were positive for *pvl* gene, while only 13% of MSSA isolates carried *pvl* gene. Also, SCC*mec* typing revealed that 8 of 10 *pvl* positive MRSA isolates had a SCC*mec* type III and 2 remaining isolates had SCC*mec* type IV and I. MLST analysis indicated that various STs harbored *pvl* gene including ST6 (SCC*mec* type IV and I), ST 239, and ST343. In one study by Havaei et al. on a population of 83 *S. aureus* isolates from Alzahra hospital, it was found that *pvl* gene was carried by ST6 and ST239 and was not detected in ST 859 (26). These findings are in agreement with our previous results and demonstrated that *pvl* positive clones of MRSA in Alzahra hospital are expanding. Moreover, the most prevalent enterotoxin gene among *S. aureus* isolates in this survey was *sea* (44%), which was lower than the findings in north of Iran (60%) (36) and Tehran (64.1%) (5) and was in agreement with another study conducted in Tehran (46.9%) (37). The second most frequent enterotoxin gene among *S. aureus* isolates was *sed* (38%), followed by *sec* and *seb* found in 21% and 17% of the isolates, respectively. Statistical analysis indicated that there was no correlation between the prevalence of toxin genes in MRSA and MSSA. In the present study, high frequency of MSCRAMMs and *ica* genes was observed. The most prevalent gene was *eno* (95%), followed by *icaD* (76%), *icaA* (73%), *fib* (64%), *cna* (60%), *ebpS* (50%), and *fnbB* (19%), but no *fnbA* gene was detected in our isolates. Furthermore, our study indicated that the higher prevalence of *icaA, icaD, cna, fib, ebpS* and *sec* genes in clinical samples, compared to nasal samples, was statistically significant. This may reflect the critical role of these genes in colonization and pathogenesis of *S. aureus* isolates.

## CONCLUSION

Our study revealed a diversity in genetic backgrounds of *S. aureus* isolates in Alzahra hospital. According to the MLST, and SCC*mec* typing, ST859, ST239, ST291, ST6, and ST25 clones with SCC*mec* type I–III are prototype clones in Iranian hospitals and our region. However, the spread of PVL positive MRSA clones can be a new challenge for clinicians. The relatively high prevalence of toxins and MSCRAMMs in *S. aureus* isolates indicate a high capacity of *S. aureus* strains in colonization and pathogenicity. Continuous *S. aureus* surveillance studies are necessary to determine the phenotypic and genotypic characteristics of the *S. aureus* clones in ALzahra hospital and other hospitals in Isfahan.
